# Can We Negotiate with a Tumor?

**DOI:** 10.1371/journal.pone.0103834

**Published:** 2014-08-01

**Authors:** Claire M. Wolfrom, Michel Laurent, Jean Deschatrette

**Affiliations:** Equipe « Dynamiques cellulaires et modélisation », Inserm Unité 757, Université Paris-Sud, Orsay, France; University of Torino, Italy

## Abstract

Recent progress in deciphering the molecular portraits of tumors promises an era of more personalized drug choices. However, current protocols still follow standard fixed-time schedules, which is not entirely coherent with the common observation that most tumors do not grow continuously. This unpredictability of the increases in tumor mass is not necessarily an obstacle to therapeutic efficiency, particularly if tumor dynamics could be exploited. We propose a model of tumor mass evolution as the integrated result of the dynamics of two linked complex systems, tumor cell population and tumor microenvironment, and show the practical relevance of this nonlinear approach.

## Introduction

The dynamics of tumor mass increase are determinant for therapeutic efficacy. Numerous mathematical models have been developed in attempts to elucidate the mechanisms underlying tumor mass dynamics. This approach is difficult because of two characteristics of tumor size increase: the variability of the dynamics, and the complexity of the causative factors.

Imaging techniques allow observations of the dynamics of tumor mass increase. The findings illustrate the wide variability of tumor doubling-times in different patients, even for a single histopathological type of tumor. Such variability has been demonstrated for lung [1], pituitary [2], liver [3], [4], brain [5], [6], prostate [7], blood [8], head and neck [9], kidney [10], [11], and breast [12]–[14] cancers. The same longitudinal studies also showed that, with the exception of very rapidly growing cancers which tend to follow exponential or Gompertz-like kinetics [15], [16], the rate of tumor progression in any one patient can vary substantially over time. For all the tumor types listed above, untreated tumor growth can vary from partial regression to no growth, to growth phases with variable rates; furthermore, these phases appear to be unpredictable [ref above and 17, 18]. Thus, fixed portraits of tumor growth are very unlikely to reflect the clinical reality.

In addition to the nonlinearity of tumor growth, the second difficulty associated with mathematical modeling of tumor growth lies in the complexity of influential factors. A host of factors in tumor cells and in the tumor cell microenvironment contribute to determining the progression of tumors. Cellular factors include rates of tumor cell death and of cell division (measured as indexes by pathologists), and also epigenetic and genetic status, including telomere repair activity [19], [20] and various driver mutations, which somehow define the degree of malignancy of tumor cells. For instance, ten subtypes of breast cancer have been described, with various genetic variants resulting in distinct tumor development profiles [21]. Variability of this type has also been shown for gastric cancer [22] and colorectal cancer [23]. The tumor cell microenvironment, defined here as all tumor constituents other than tumoral cells, can both restrain and promote tumor growth, and the equilibrium between the two effects is variable [24], [25]. The microenvironment includes biochemical factors such as local concentrations of oxygen [26]–[29], nutrients [30]–[33], and H+ ions [34]–[36], physical features such as matrix density [37] and vascularization [38], immunological defenses [39], [40], and the various different cell types and their relative proportions in the tumor [41]. These microenvironmental factors are all difficult to quantify, vary considerably both between tumors and between parts of any single tumor [42], and display dynamic and unpredictable changes. This complexity has been translated into increasingly complicated models, which, however, seldom correspond well to observations made by physicians and radiologists. We propose that a better approach to the spontaneous irregularity of growth of most malignancies would be nonlinear analysis and modeling, and that this approach may have clinical applications.

## Model and Methods

### Model of nonlinear tumor growth

In view of the practical considerations described above, we chose to use a novel approach to modeling tumor growth. We considered the evolution of tumor mass as the net result of interplay between two complex systems: a “tumor cells” system (Cell) and a “tumor cell environment” system (Env). Clinical observations indicate that: both systems oscillate with marked and unpredictable irregularities; their components are nevertheless strongly determined by various feedback and feedforward controls; and the two systems are linked to each other. These properties are characteristic of coupled chaotic oscillatory systems. They also imply that tumor mass evolution will depend upon the integration of the dynamics of these two systems (Cell and Env).

Various types of mathematical oscillators, initially describing physical measures, have been used to model systems with similar characteristics.

**Figure 1:**
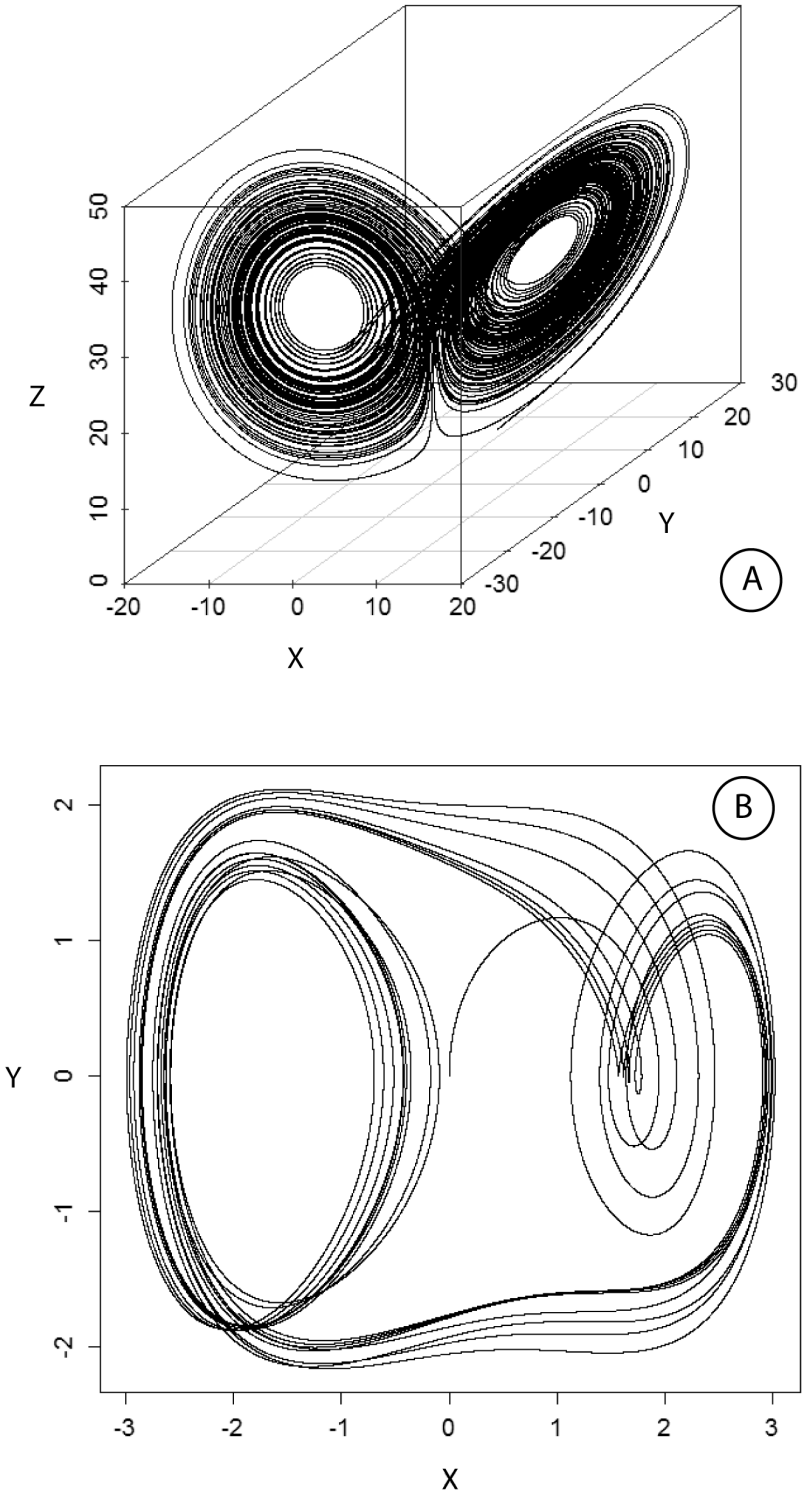
Phase plane representation of the uncoupled Lorenz-Cell oscillator (Fig. A) and the Duffing-Env oscillator (Fig. B). For the Cell oscillator, parameter p_1_ is constant (p_1_ = 10) indicating the absence of coupling between cellular and environmental oscillators. Parameters and equations are as indicated in the *Model and methods* section.

The rationale for the choice of the “Cell” oscillator was as follows: **i**) a two-well oscillator was selected because our previous work on chaotic-like oscillations of tumor and progenitor cell proliferation, *in vitro* and *in vivo*, had shown a balance between high/low fixed points [43]–[45]; and **ii**) the level of complexity of the oscillator required at least three linked variables to reflect interplay between three critical and complex mechanisms which control a cell population: cell death, which varies greatly in some tumors [46]–[48], cell proliferation which fluctuates, and genetic status, including telomere repair [19], [43] and gene expression, which displays oscillations [48], [49]. The three-variable Lorenz oscillator was adapted to these constraints, and was used to illustrate the “Cell” oscillator (Figure 1A), which was written thus:
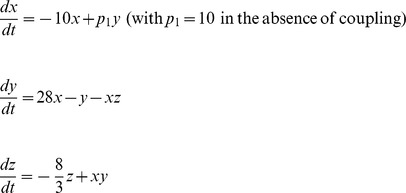



The rationale for the choice of the “Env” oscillator was as follows: **i**) a two-well oscillator was selected to reflect the balance between the enhancing and inhibitory effects of the tumor cell environment; **ii**) the oscillator had to include both a damping term reflecting soluble and immune defenses, and a restoring force reflecting autostimulatory effects of tumor cells and the tumor matrix [24], [50], [51]; **iii**) periodicity had to be introduced into the oscillator to reflect the net influence of metabolic and hormonal clocks [52]–[54]. The classical Duffing oscillator including a periodic external forcing is adapted to these constraints and was therefore chosen as the “Env” oscillator (Figure 1B), written thus:
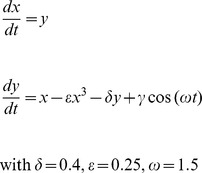
where εx^3^ is the restoring force of the system, δy is the damping force, and γ cos(ωt) is the periodic external forcing.

The two oscillators were then coupled, to reflect the reciprocal influences of the dynamics of the tumor cell population and the dynamics of the microenvironment. Synchronization was obtained using parameter p_1_ of the Cell oscillator proportional to the y or x variable of the Env oscillator (p_1_ = 100 y_env_ or p_1_ = 100 x_env_). Our hypothesis was that the integrated signal of the two coupled oscillatory systems would result in waves of tumor growth at times of synchronized maxima of each oscillator. In both equations, we purposely kept standard values of variables and parameters responsible for chaotic behavior of the two oscillators. Although unrelated to biological numbers, the use of these values is coherent with our general approach.

### Integrated signal and external control of the coupled oscillators

Our next step was to interfere with the oscillators to test how we could curb the integrated signal reflecting tumor mass increase. By analogy with what occurs in clinical practice, the interference with the Cell oscillator would illustrate the effects of chemotherapy, which directly induces tumor cell death, and the interferences with the Env oscillator would illustrate the effects of various adjuvant treatments. In general, progressive control of a Duffing oscillator requires at least one of three actions: increasing the damping effect [55], decreasing amplitude of the restoring force, or adjusting the frequency and amplitude of periodic external driving [56]. Therefore, we examined how changes in these three phenomena changed the synchronization of the two systems.

## Results

### Synchronization of the two oscillators

**Figure 2:**
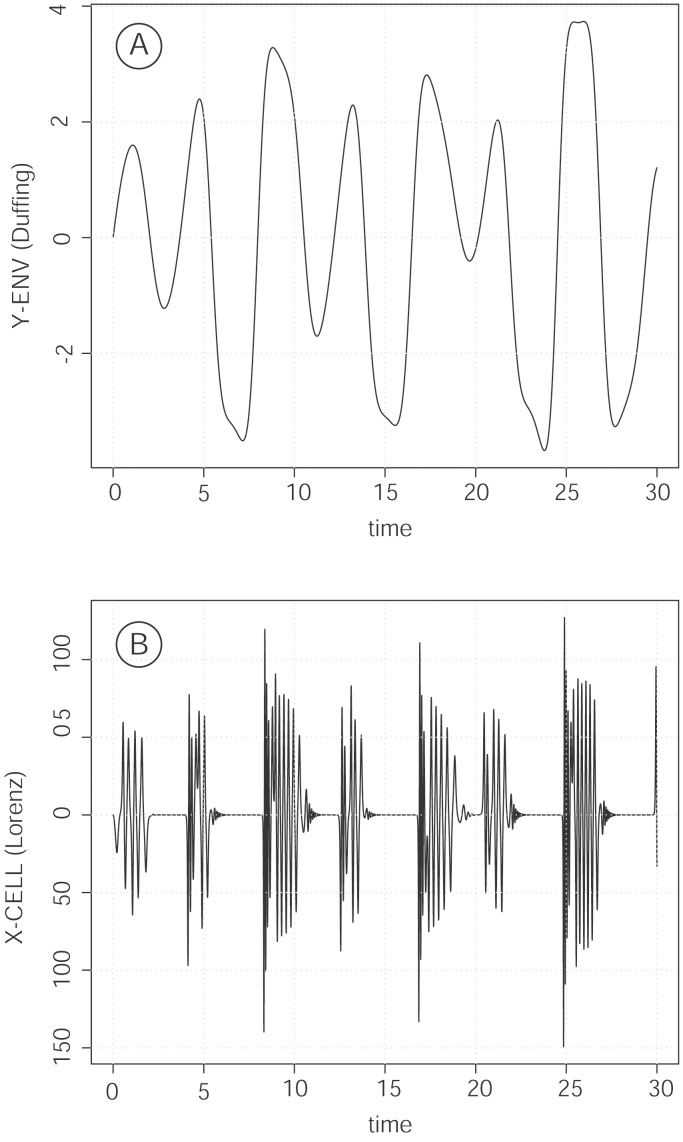
Burst oscillations result from the coupling of the Env oscillator (Fig. 2A) and the Cell oscillator (Fig. 2B). Coupling was obtained through the p_1_ parameter in the Cell oscillator, by setting p_1_ = 100 y_env_, where y_env_ is the y variable of the Env oscillator. The data shown represent the changes through time of the y variable of the master, Env oscillator (Fig. 2 A) and of the x variable of the coupled Cell oscillator (Fig. 2 B). Parameters and equations are as indicated in the *Model and methods* section.

**Figure 3:**
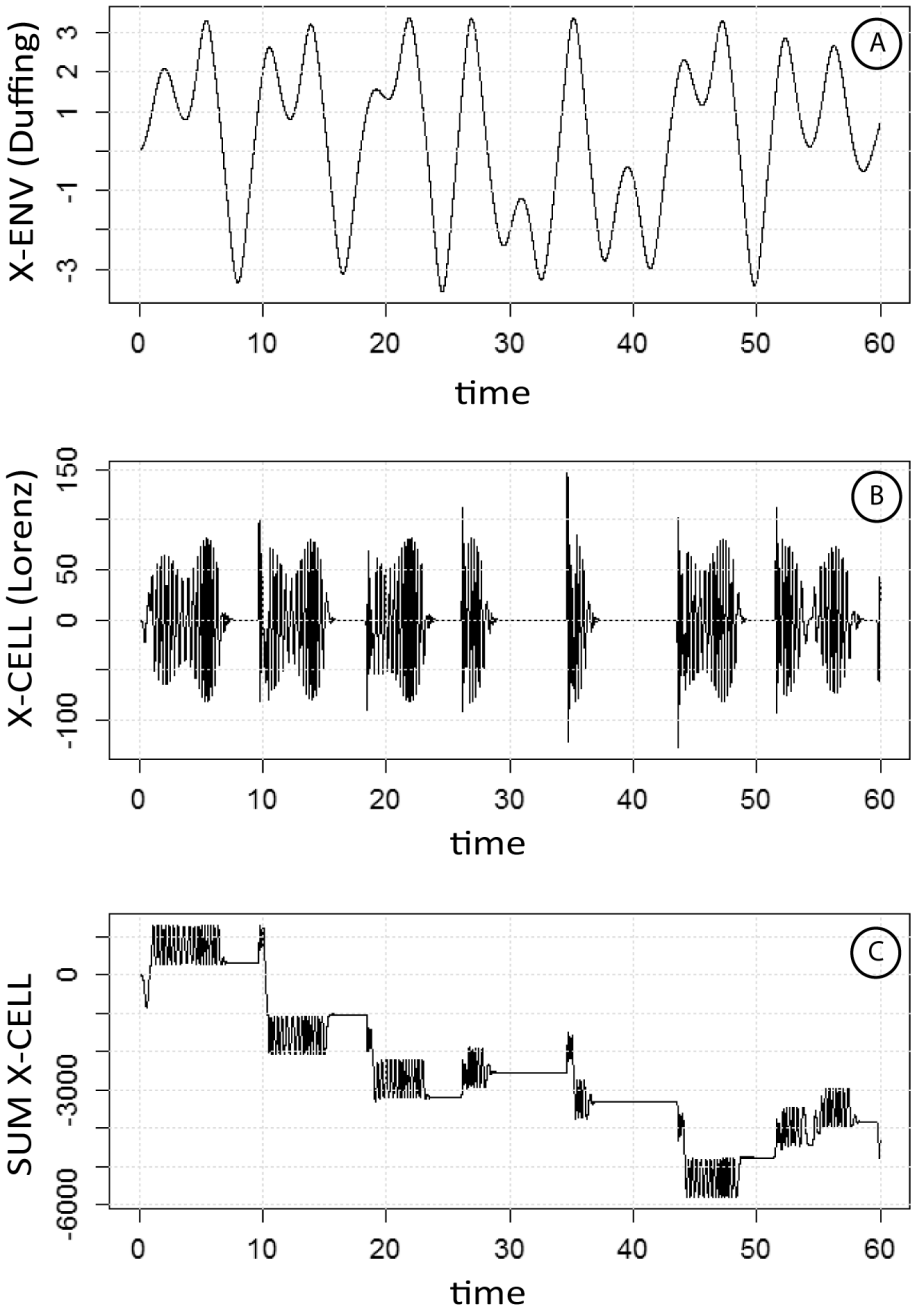
Bursting is observed by coupling the Cell oscillator to either of the two variables of the Env oscillator (Fig. 3A,B,C). Unlike the model in Figure 2, coupling is obtained by setting p_1_ = 100 x_env_, where x_env_ is the first variable of the Env oscillator. C: cumulated signal of the synchronized Cell oscillator.

Phase locking of the two oscillators was obtained directly using parameter p_1_ of the Cell oscillator proportional to the variable y of the Env oscillator, while the amplitudes of the two systems remained variable and uncorrelated. As a result of synchronization, the Cell oscillator showed grouped bursts of fluctuations, strictly linked to ascending segments of oscillations of the Env oscillator (Figure 2A and B). The coupling was very robust, and was observed with similar strength when any one of the three variables of the Cell oscillator was used for coupling. Synchronization was also obtained using another type of coupling, such as p_1_ proportional to the variable x of the Duffing equation, and again the Cell oscillator displayed bursts of fluctuations linked to the peaks of the controlling Env oscillator. However, the Cell oscillator was entrained only by peaks corresponding to positive values of the variable x of Env, or, in other words, the right well of the Env oscillator. The Env left well did not affect the activity of the Cell oscillator (Figure 3A and B). Changes in coupling intensity by increasing the values of parameter p_1_ (to 10, 50, 100, or 1000) resulted in increased numbers of harmonics in each burst of the Cell oscillator. However, synchronization remained identical and neither the onset nor the length of bursts were affected (data not shown).

### Integrated signal and external control of the coupled oscillators

The integrated signal from the Cell oscillator synchronized with the Env oscillator was alternating irregular ascending or descending staircase segments. The slope of the signal varied according to sampling intervals, a direct consequence of the classical dependence of chaotic oscillators on initial conditions. This signal was clearly consistent with the fluctuating evolution of tumor mass, displaying increases with variable slope, with phases of stability and partial regressions (Figure 3 C). However, to predict the long-term net result of the activity of the whole system, which illustrates the progression of tumor mass, the total length of the silencing intervals of the Cell oscillator appear to be particularly significant: the value of this length is not dependent on the conditions of integration.

### External control

**Figure 4:**
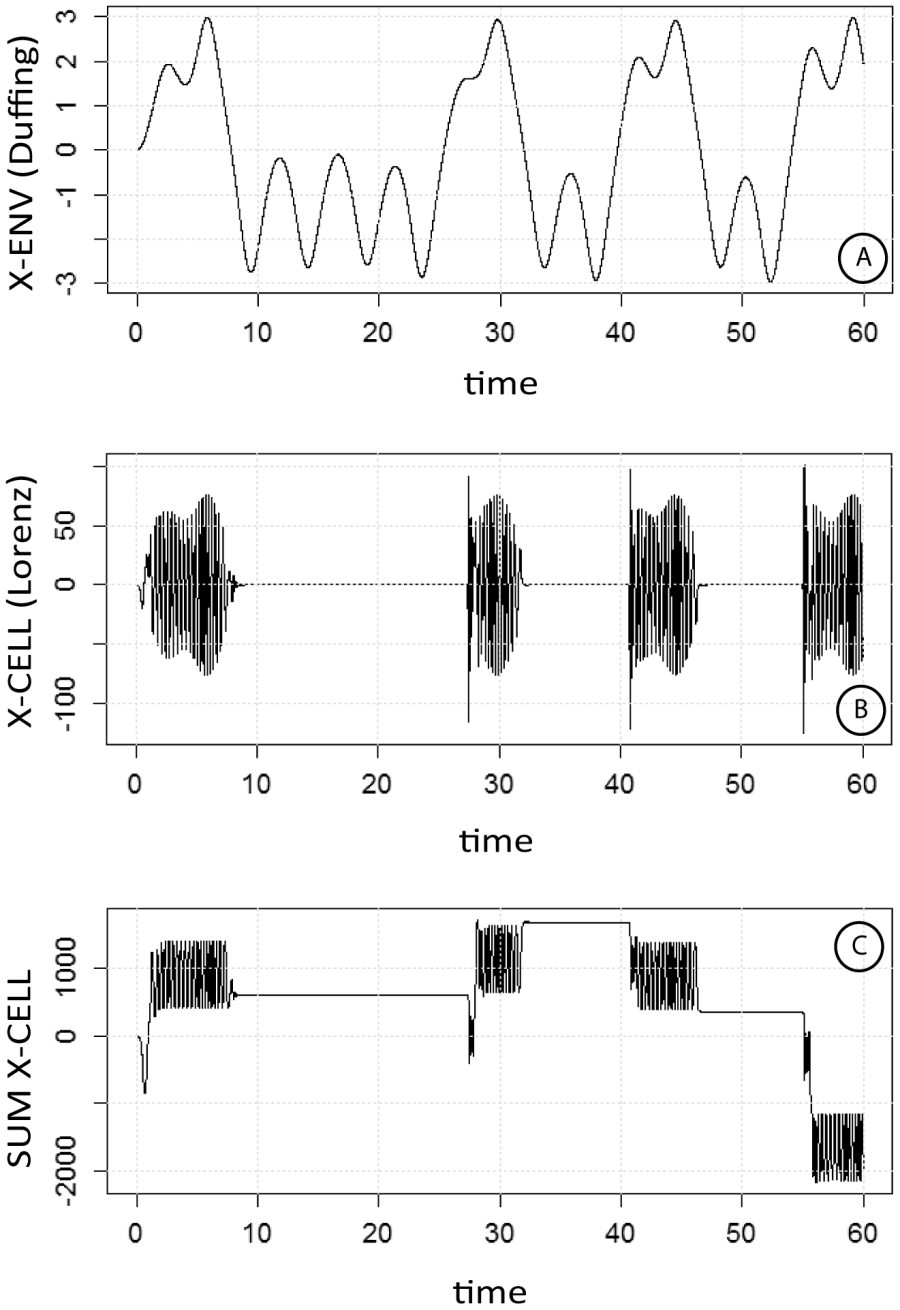
Influence of the pattern of evolution of the Env oscillator (Fig. A) on the response of the coupled Cell oscillator (Fig. B, C): effects of γ and ω. As in Figure 2, except that the changes through time of the Env oscillator were modified by making γ = 1.5 and ω = 1.3 (in place of 2.5 and 1.5, respectively). As a consequence, the Env oscillator exhibited irregularly alternating large (positive x zone) and small peaks (negative x zone). Under these conditions, the coupled Cell oscillator only responded to large peaks and did not respond to small peaks. C: cumulated signal of the synchronized Cell oscillator.

**Figure 5:**
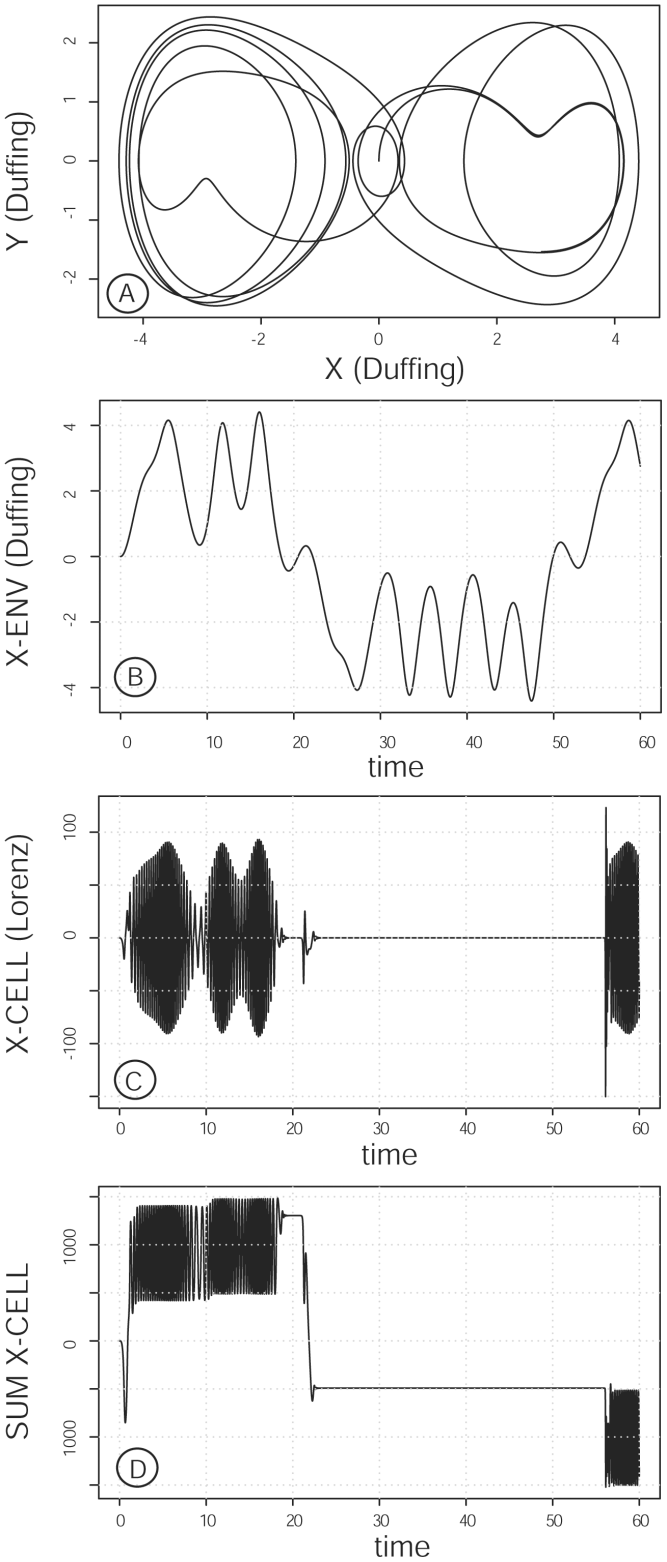
Influence of changes in the pattern of evolution of the Env oscillator (Fig. A) on the response of the coupled Cell oscillator (Fig. B, C): role of ε. As in Figure 2, except that the changes through time of the Env oscillator were modified by making ε = 0.1 (in place of 0.25). As a consequence, the Env oscillator exhibited grouped large (positive x) peaks alternating with grouped small (negative x) peaks. Under these conditions, the coupled Cell oscillator only responded to bundles of large peaks. C: cumulated signal of the synchronized Cell oscillator displaying prolonged phases of silencing.

- We tested the consequences of a smaller amplitude (the value of parameter γ = 1.5 rather than 2.5) and lower frequency (parameter ω = 1.3 rather than 1.5) of the external periodic force of the Env oscillator. This resulted in irregular alternation between positive and negative peaks of the Env oscillator, with increased frequency of peaks in the left well of the Env oscillator (negative x). Under these conditions, the coupled Cell oscillator was insensitive to negative x Env peaks and was only entrained by positive x values (Figure 4 A, B,C).
- An increase in the restoring force of the Env oscillator (that is an increase of ε to 0.5), changed the form of oscillations, which became more periodical, displaying large regular peaks, with perfectly synchronized bursts of the Cell oscillator (data not shown). In contrast, a decrease of ε to 0.1 resulted in alternating zones of positive and negative peaks of the Env oscillator, and corresponding zones of low amplitude bursts and prolonged silence of the Cell oscillator (Figure 5).
- An increase in parameter δ made the basal motif of the Env oscillator more complex with the emergence of shouldering of the peaks. However, the Cell oscillator bursts responded to each of the x-positive Env peaks. In contrast, a decrease in δ made the Env oscillations simpler, and again the Cell oscillator responded to each x-positive peak of the leading Env oscillator (data not shown).

## Discussion

We show in this analysis that two linked chaotic systems, images of the tumor cell population and tumor cell environment, respectively, are readily and solidly synchronized. As a result, all large positive peaks of the Env oscillator, which correspond to the positive x domain in the phase space portrait, entrained bursts of the Cell oscillator. When changes in parameters led to the peaks of the Env oscillator being in the negative domain, the coupled Cell oscillator was inactive. The integrated signal resulting from this synchronization was an irregular staircase curve, a profile consistent with the waves of tumor cell proliferation as commonly observed *in vivo*: waves of progressive irregular increases of tumoral mass interspersed with plateaus and partial regressions.

The irregular growth of many malignancies has consequences for therapy. In particular, therapeutic inefficacy is likely during phases where there is either no net tumor growth or tumor regression, raising issues about the overtreatment of some tumors as recently discussed [57]–[59]. A new step in the personalization of treatments involving adapting therapy time-schedules to the velocity of tumor growth may be beneficial. This would require considering the tumor as a mostly chaotic system in which “initial conditions” (i.e. the net energy for growth) changes constantly. Various random variations must necessarily be included in this complex system of feedback controls, as in all physiological systems. These complex dynamics result in inter- and intra-individual variability, making the prediction of tumor growth phases impossible; nevertheless, the detection of circulating tumor cells [60] and sequential imaging [61]–[65] can be used for regular monitoring of tumor mass evolution. An adapted therapeutic approach would tend to control, progressively, the complex tumor system rather than eradicating it in one step. This strategy was recently proposed by Gatenby *et al* using ovarian cancer cells grown in SCID mice: the so-called “adaptive therapy” persistently controlled, and in some cases finally suppressed, the tumors, with minimal toxicity and prolonged mouse survival. This therapy involved treatment with small doses of carboplatin, only when a tumor increased in size, but did not involve trying directly to eliminate it [40], [66], [67]. The initial goal of these authors was to allow chemosensitive cells to survive so that they limit the proliferation of resistant cells. Also, prolonged intervals between treatments allow some recovery of cell chemosensitivity [68], [69]. Our interpretation is that tailoring treatment to the irregular dynamics of tumor growth also supported physiological control. According to our model of synchronized “tumor cell” and “tumor cell environment” oscillators, the various changes in parameters which influence the integrated signal may find analogy in three types of actions, all of which have some degree of antitumor effect in clinical practice. The first action is “adaptive therapy” to destroy newly proliferating cells, thereby decreasing what we refer to above as the restoring force of the tumor, so that every growth phase of the tumor is opposed to by proportional chemotherapy. Second, increased damping in the tumor cell microenvironment is very similar to what results from persistent multidisciplinary support such as moderate use of hyperoxia [70], [28], [29] and systemic buffers [35], [36], glucose metabolism control [71], [72], and immunity enhancement [72], [73]. In particular, immunity can maintain cancer cells in a dormant state [40], [74]. The third interference would be to regularize the periodic stimulations affecting cell proliferation. The antitumor effects of circadian re-entrainment by light and meal-timing in murine models illustrate this point well [52], [53]. Extratumoral periodic forces also include hormonal clocks [75]–[78], which should be considered in their rhythmic pattern.

A further possible advantage of “negotiating” with the tumor according to the phases of its dynamics is to avoid the boomerang-effect of tumor mass eradication, which frequently induces compensatory growth of both the residual tumor cells [79]–[82] and of the frequent and early occurring dormant micrometastases [83]–[85].

Clearly, adapted randomized trials in models are required if sufficient evidence is to be obtained to validate this negotiating approach. However, there are potential clinical benefits of developing some guerilla strategies for overcoming the nonlinearity of tumor growth.
